# The association between social vulnerability and racial disparities in preterm birth

**DOI:** 10.1002/pmf2.70279

**Published:** 2026-04-07

**Authors:** Anita Pershad, Gabby Adams, Lindsay Robbins, George Saade, Tetsuya Kawakita

**Affiliations:** ^1^ EVMS Department of Obstetrics and Gynecology Macon & Joan Brock Virginia Health Sciences Eastern Virginia Medical School at Old Dominion University Norfolk Virginia USA; ^2^ EVMS Center for Maternal and Child Health Equity and Advocacy Macon & Joan Brock Virginia Health Sciences Eastern Virginia Medical School at Old Dominion University Norfolk Virginia USA

**Keywords:** health disparities, preterm birth, Social Vulnerability Index

## Abstract

**Objective:**

To evaluate the association between county‐level social vulnerability and preterm birth and assess whether racial disparities vary across levels of vulnerability.

**Methods:**

This cross‐sectional study utilized restricted Centers for Disease Control and Prevention natality data from 2023. We included Black or White births with singleton pregnancies across 3123 US counties from 50 US states and the District of Columbia. Participants were grouped into quartiles based on the county‐level Social Vulnerability Index (SVI), with quartile 4 representing the most socially vulnerable populations. The primary outcome was preterm birth, defined as delivery before 37 weeks. Secondary outcomes included small for gestational age (SGA; birth weight <10th percentile) and hypertensive disorders of pregnancy (HDP). We used mixed‐effects generalized linear models with Poisson distribution with robust variance to calculate average marginal effects (AMEs) with 95% confidence intervals (95% CIs), adjusted for confounders. Difference‐in‐differences (DID) was calculated for the difference in Black–White disparity across SVI quartiles, with quartile 1 as a reference.

**Results:**

A total of 2,122,789 births were included: 355,774 in quartile 1; 535,907 in quartile 2; 611,074 in quartile 3; and 620,034 in quartile 4. Black births had higher adjusted rates of preterm birth than White births in all quartiles. Black and White preterm birth rates were 9.8% versus 7.8% (AME, 2.0%; 95% CI, 1.6–2.5) in quartile 1; 11.0% versus 7.9% (AME, 3.1%; 95% CI, 2.7–3.4) in quartile 2; 11.3% versus 8.3% (AME, 3.0; 95% CI, 2.–3.3) in quartile 3; and 12.2% versus 8.6% (AME, 3.7%; 95% CI, 3.4–4.0) in quartile 4. Racial disparities increased with higher social vulnerability; DID was statistically significant in quartiles 2–4 (1.1% [95% CI, 0.5–1.6] in quartile 2, 1.0% [95% CI, 0.4–1.6] in quartile 3, and 1.6% [95% CI, 1.1–2.2] in quartile 4 when compared to quartile 1). Similar patterns were observed for SGA and HDP.

**Conclusion:**

Increasing social vulnerability was associated with widening racial disparities in preterm birth and other adverse pregnancy outcomes. These findings underscore the need to address community‐level influences on perinatal inequities.

## 1 INTRODUCTION

Preterm birth remains a leading cause of neonatal morbidity and mortality in the United States and contributes substantially to long‐term health inequities [1, 2]. Despite advances in obstetric and neonatal care, Black birthing individuals consistently experience higher rates of preterm birth compared with White individuals, a disparity that has persisted for decades [3, 4]. While individual‐level factors, such as maternal age and medical comorbidities, contribute to risk, mounting evidence indicates that structural and community‐level conditions, including poverty, systemic racism, and environmental exposures, are critical drivers of perinatal health outcomes [2, 3, 4].

The Centers for Disease Control and Prevention (CDC) Social Vulnerability Index (SVI) is a composite indicator designed to measure community capacity to withstand and recover from external stressors, such as natural disasters and public health crises [5]. The SVI incorporates domains including socioeconomic status, household composition, minority status and language, housing, and transportation. Originally developed for disaster preparedness, the index is increasingly applied in maternal and child health research as a proxy for neighborhood disadvantage [6, 7].

Prior studies have demonstrated associations between higher SVI and adverse outcomes such as preterm birth, low birth weight, and maternal morbidity [8, 9, 10, 11, 12, 13, 14]. However, whether the magnitude of racial disparities in preterm birth is consistent within and across levels of social vulnerability remains unclear. Understanding how community‐level disadvantage modifies racial inequities is essential for guiding targeted policy and public health interventions.

We sought to examine the association between county‐level SVI and preterm birth and assess whether Black–White disparities varied across levels of community‐level vulnerability. We hypothesized that preterm birth rates would be higher in more vulnerable counties and that disparities between Black and White birthing people would widen as vulnerability increased.

## 2 MATERIALS AND METHODS

This cross‐sectional study analyzed de‐identified county‐level live‐birth data on births in the United States, including 50 states and the District of Columbia, in 2023 [15]. The restricted‐use natality dataset was accessed through the CDC under an approved research proposal. The year 2023 was selected because it represented the most recent data available at the time of analysis.

The study population included all singleton births at 22 weeks’ gestation or greater. To specifically evaluate racial disparities between Black and White births, analyses were restricted to self‐identified non‐Hispanic Black and non‐Hispanic White births, classified according to CDC standardized categories of race and ethnicity. Exclusions included cases with fetal anomaly or missing information on gestational age or county‐level SVI. Fetal anomalies captured in the dataset included anencephaly, spina bifida, cyanotic congenital heart disease, congenital diaphragmatic hernia, omphalocele, gastroschisis, cleft lip and/or palate, hypospadias, limb deficiencies, and chromosomal disorders. Chromosomal disorders were defined as trisomy 21 or any group of birth defects associated with known chromosomal abnormalities. Gestational age was based on the best obstetric estimate as reported on the birth certificate. This study qualified for exemption from Institutional Review Board review and adhered to the STROBE reporting guidelines.

The primary exposure was county‐level social vulnerability, measured using the 2022 CDC SVI [8, 16]. The SVI integrates 16 US Census–based indicators across four domains: (1) socioeconomic status (poverty, unemployment, housing cost burden, lack of high school diploma, no health insurance), (2) household composition (age ≥65 years, age ≤17 years, disability, single‐parent household, limited English proficiency), (3) minority status, and (4) housing/transportation (multiunit housing, mobile homes, overcrowding, no vehicle access, group quarters) [5]. Values range from 0 to 1, with higher scores reflecting greater vulnerability. For analysis, overall and domain‐specific SVI values were categorized into quartiles: quartile 1 (SVI 0–0.25; least vulnerable), quartile 2 (SVI 0.26–0.5), quartile 3 (SVI 0.51–0.75), and quartile 4 (SVI 0.76‐1.0; most vulnerable).

The primary outcome was preterm birth, defined as delivery <37 weeks’ gestation. Secondary outcomes included: (1) small for gestational age (SGA), defined as birthweight <10th percentile for gestational age and sex [14], (2) hypertensive disorders of pregnancy (HDP), defined as gestational hypertension, preeclampsia, or eclampsia. HDP age was defined as birthweight below the 10th percentile for gestational age and sex using the 2017 US singleton birthweight reference based on obstetric estimates of gestation [16]. HDPs were defined based on birth certificate reporting of gestational hypertension, which encompasses hypertensive conditions diagnosed during pregnancy.

### 2.1 Statistics

Demographic and clinical characteristics were compared across SVI quartiles using χ^2^ tests for categorical variables and one‐way ANOVA for continuous variables. To quantify the difference in disparity in the rate of adverse outcomes across the SVI quartiles, we fitted modified Poisson regression models with robust standard errors [16]. We included race‐by‐SVI interaction terms to examine whether the relationship between social vulnerability and adverse outcomes varied by race. Covariates were chosen based on the prior literature regarding the association with adverse pregnancy outcomes and included maternal age, prepregnancy body mass index, insurance type, smoking status, chronic hypertension, and pregestational diabetes [17, 18, 19, 20, 21, 22, 23]. To account for clustering at the county and state levels, we also included the random effects of the county and state. Adjusted rates of outcomes were calculated using the marginal standardization form of predictive margins according to race [18]. Average marginal effects (AMEs) and 95% confidence intervals (CIs) were estimated for outcomes by race within each quartile. Difference‐in‐differences (DID) analyses tested whether racial disparities in outcomes were greater in quartiles 2–4 compared with quartile 1. Interaction *p* values were calculated using the likelihood‐ratio test.

### 2.2 Sensitivity analysis

We conducted several sensitivity analyses to test the robustness of our findings. First, to examine whether the association between SVI and outcomes was linear, we modeled SVI using restricted cubic splines with five knots placed at the 5th, 27.5th, 50th, 72.5th, and 95th percentiles of the SVI distribution, following Harrell's recommended percentiles. Nonlinearity was evaluated by jointly testing the coefficients of the nonlinear spline terms using a Wald test; a nonsignificant result indicating no statistical evidence of deviation from linearity. For the outcomes with nonlinear association, we used the marginal standardization form of predictive margins and plotted adjusted rates according to race and SVI. Second, for outcomes demonstrating a linear association with SVI, we evaluated the interaction between continuous SVI and race to assess potential effect modification. Treating SVI as a continuous variable allows for assessment of a dose–response relationship across the full spectrum of social vulnerability, rather than relying solely on comparisons between arbitrarily defined quartiles. We fitted modified Poisson regression models to calculate adjusted relative risks (aRRs) per one interquartile range (IQR) increase in SVI with 95% CI, controlling for the above‐mentioned covariates. Predicted rates of outcomes were plotted using the above‐mentioned method. Third, because the clinical consequences of preterm birth intensify with decreasing gestational age, we repeated the analysis using different severities of preterm birth (<34, <32, and <28 weeks). Fourth, we repeated the analysis after excluding counties with <100 births in either racial group. Statistical significance was defined as *p* < 0.05. Analyses were performed using Stata 18.5 (StataCorp).

## 3 RESULTS

**FIGURE 1:**
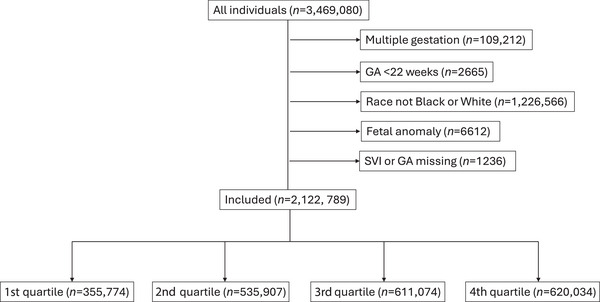
Flowchart of study population. GA, gestational age; SVI, Social Vulnerability Index.

**FIGURE 2:**
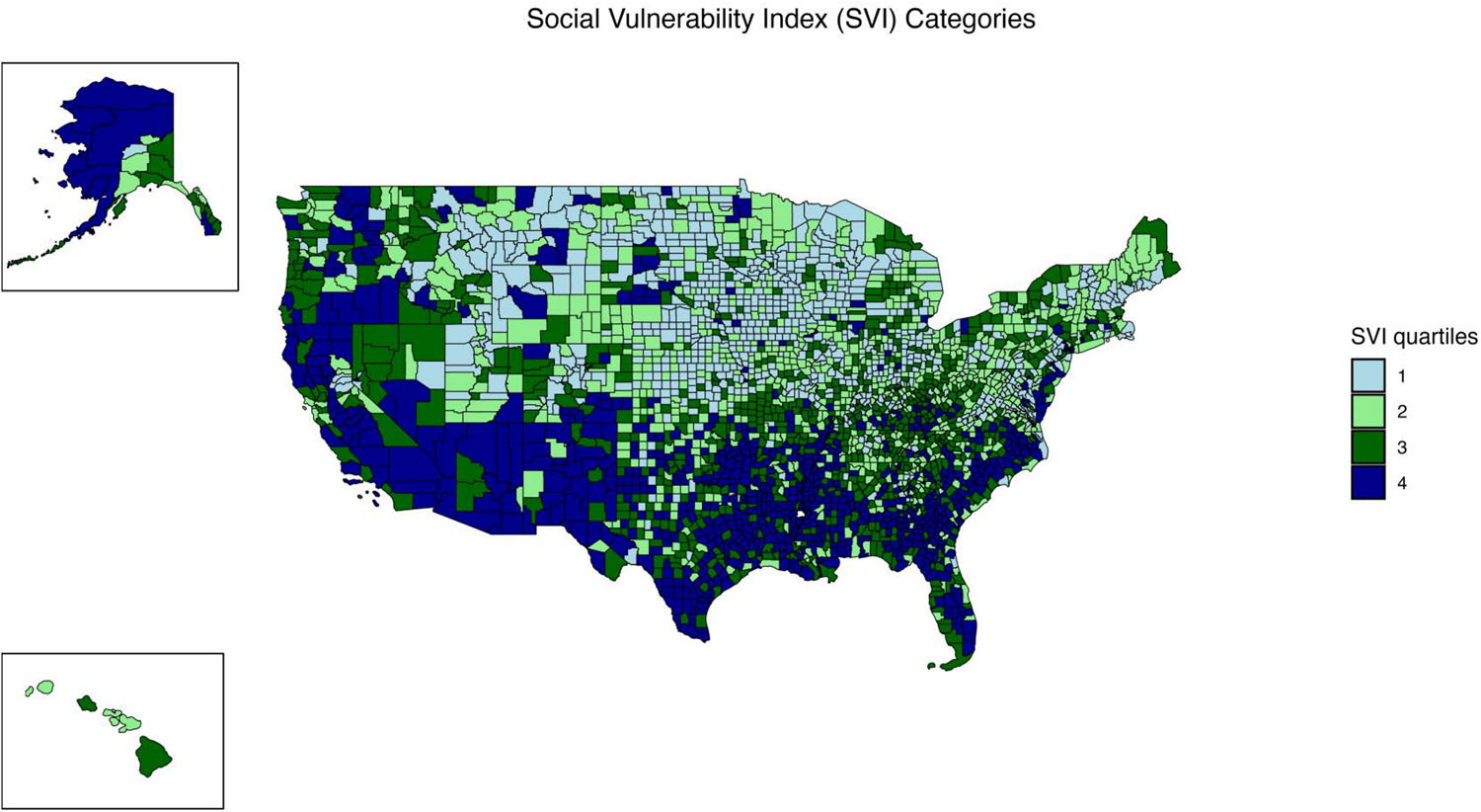
Geographic distribution of Social Vulnerability Index (SVI) quartiles across US counties. This map displays all US counties categorized into quartiles of the CDC SVI, with quartile 1 (lightest shade) representing the least vulnerable communities and quartile 4 (darkest shade) representing the most socially vulnerable communities. Counties in the highest SVI quartile were predominantly concentrated in the South and parts of the West, while lower‐vulnerability counties were more common in the Midwest and Northeast. Alaska and Hawaii are shown in insets for completeness. CDC, Centers for Disease Control and Prevention; GA, gestational age.

We included a total of 2,122,789 births from 3123 counties: 355,774 in quartile 1, 535,907 in quartile 2, 611,074 in quartile 3, and 620,034 in quartile 4 (Figure 1). The geographic distribution of SVI quartiles is illustrated in Figure 2. Counties in the highest, most vulnerable SVI quartile (dark blue) were primarily concentrated in the South, Southwest, and Alaska, whereas those in the lowest, least vulnerable quartile (light blue) were predominantly located in the Midwest and Northeast. Demographic characteristics stratified by SVI quartile and race are presented in Table S1; all demographic variables differed significantly across groups.

Outcomes stratified by race and SVI are presented in Table 1. The prevalence of preterm birth increased with higher levels of social vulnerability for both Black and White births. Among Black births, preterm birth rates rose from 9.82% (95% CI, 9.34–10.30) in the lowest SVI quartile (Q1) to 12.23% (11.94–12.52) in the highest quartile (Q4). Among White births, rates increased from 7.79% (7.63–7.94) in Q1 to 8.55% (8.37–8.74) in Q4. The AME comparing Black and White births widened from 2.03% (1.56–2.50) in Q1 to 3.68% (3.36–3.99) in Q4. The DID also increased across quartiles, from reference in Q1 to 1.64% (1.08–2.20) in Q4. The interaction between race and SVI was statistically significant (interaction *p* < 0.001), indicating that higher social vulnerability was associated with a greater racial disparity in preterm birth. Similar patterns were observed for HDP and SGA, although the DID for Q2 was not statistically significant, and the interaction term for SGA did not reach statistical significance. Crude analyses incorporating random intercepts for state and county are presented in Table S2. Results were generally consistent with the primary analysis, except that the interaction term for SGA reached statistical significance.

**TABLE 1:** Outcomes according to the Social Vulnerability Index (SVI) and race.

Outcome	SVI quartile	Black (%, 95% CI)	White (%, 95% CI)	AME (95% CI)	DID (95% CI)	Interaction *p*
Preterm birth	Q1	9.82 (9.34, 10.30)	7.79 (7.63, 7.94)	2.03 (1.56, 2.50)	[Reference]	
	Q2	11.01 (10.64, 11.37)	7.93 (7.79, 8.07)	3.08 (2.73, 3.43)	1.05 (0.46, 1.63)	
	Q3	11.31 (11.00, 11.63)	8.28 (8.13, 8.43)	3.03 (2.72, 3.34)	1.00 (0.44, 1.55)	<0.001
	Q4	12.23 (11.94, 12.52)	8.55 (8.37, 8.74)	3.68 (3.36, 3.99)	1.64 (1.08, 2.20)	
HDP	Q1	11.31 (10.65, 11.97)	11.58 (11.28, 11.88)	−0.27 (−0.90, 0.37)	[Reference]	
	Q2	12.08 (11.47, 12.68)	11.65 (11.34, 11.96)	0.42 (−0.14, 0.98)	0.69 (−0.15, 1.53)	
	Q3	12.79 (12.29, 13.28)	11.81 (11.52, 12.10)	0.97 (0.49, 1.46)	1.24 (0.43, 2.05)	<0.001
	Q4	13.02 (12.43, 13.60)	11.21 (10.87, 11.56)	1.81 (1.15, 2.47)	2.07 (1.18, 2.97)	
SGA	Q1	13.02 (12.41, 13.63)	6.62 (6.47, 6.76)	6.40 (5.79, 7.02)	[Reference]	
	Q2	14.22 (13.80, 14.63)	7.07 (6.94, 7.20)	7.14 (6.73, 7.56)	0.74 (−0.00, 1.48)	
	Q3	14.52 (14.17, 14.86)	7.11 (6.99, 7.24)	7.41 (7.05, 7.76)	1.00 (0.29, 1.71)	0.36
	Q4	15.17 (14.90, 15.44)	7.52 (7.38, 7.66)	7.65 (7.35, 7.95)	1.25 (0.56, 1.93)	

*Note*: Numbers are models are adjusted for maternal age, body mass index, chronic hypertension, pregestational diabetes, smoking status, insurance type, and the random effects of state and county.

Abbreviations: AME, average marginal effect; CI, confidence interval; DID, difference‐in‐differences; HDP, hypertensive disorders of pregnancy; SGA, small for gestational age.

**FIGURE 3:**
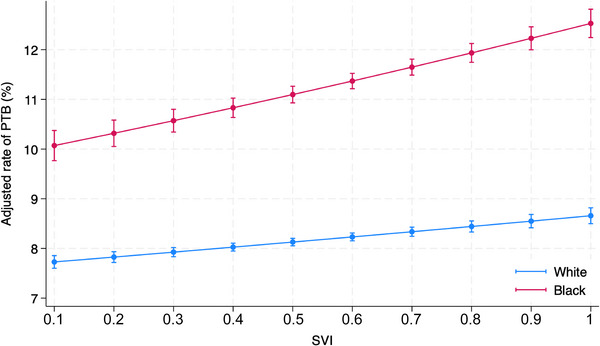
Adjusted rates of preterm birth (PTB) by Social Vulnerability Index (SVI) quartile and maternal race. This figure illustrates the adjusted rate of PTB among births to Black and White individuals across SVI quartiles, with higher quartiles indicating greater social vulnerability. PTB rates increased steadily with rising SVI for both racial groups. Across all quartiles, births to Black individuals had substantially higher adjusted PTB rates than those to White individuals, and the disparity widened in the highest SVI quartile. Error bars represent 95% confidence intervals.

For preterm birth and HDP, the associations with SVI were linear (*p* = 0.24 and *p* = 0.08, respectively), whereas the association with SGA was nonlinear (*p* = 0.005). Nonlinear association between SVI and SGA is visualized in Figure S1. When SVI was treated as a continuous variable, both higher SVI and Black race were associated with an increased risk of preterm birth (aRR per IQR [SVI], 1.07; 95% CI, 1.05–1.08 and 1.29; 95% CI, 1.23–1.34 [Black], respectively). The interaction between continuous SVI and race was statistically significant (*p* < 0.001), indicating that the association between race and preterm birth differed across levels of social vulnerability. As illustrated in Figure 3, the disparity in preterm birth between Black and White births widened with increasing SVI. For HDP, neither SVI nor race was significantly associated with risk (aRR per IQR [SVI], 0.98; 95% CI, 0.95–1.00 and 0.95; 95% CI, 0.90–1.00 [Black], respectively); however, the interaction between continuous SVI and race was statistically significant (*p* < 0.001), suggesting that the racial disparity in HDP also increased with higher social vulnerability (Figure S2).

Data on early preterm births stratified by race and SVI are presented in Table S3. Across all SVI quartiles, Black births had higher rates of preterm birth before 34, 32, and 28 weeks of gestation compared with White births. The racial disparity widened with increasing social vulnerability for preterm birth before 34 and 32 weeks, except for quartile 2 in the <32‐week group (*p* for interaction < 0.001 and 0.001, respectively). In contrast, the DID estimate for preterm birth before 28 weeks was not statistically significant (*p* for interaction = 0.36). Finally, analyses excluding counties with fewer than 100 births in either racial group yielded results consistent with the primary analysis (Table S4).

## 4 DISCUSSION

### 4.1 Principal findings

In this national cohort of more than two million births, higher county‐level social vulnerability was associated with both elevated rates of preterm birth and widening disparities between Black and White births. Although racial disparities were evident across all quartiles of vulnerability, they were substantially larger in the most disadvantaged counties. Increasing social vulnerability corresponded to widening racial gaps for multiple adverse pregnancy outcomes, with the strongest associations observed for preterm birth and HDP. These findings suggest that social disadvantage amplifies existing inequities in maternal health. By contrast, the interaction between race and the SVI for SGA birth was not statistically significant.

### 4.2 Summary of findings in the context of the literature

Our findings are consistent with a substantial body of literature linking community disadvantage to adverse perinatal outcomes, including preterm birth, low birth weight, and maternal morbidity [8, 9, 10, 11, 12, 13, 14]. Prior studies show that higher social vulnerability, reflecting economic, educational, housing, and healthcare inequities, is associated with elevated risks of these outcomes [8, 9, 10] and that these risks disproportionately affect Black birthing people, particularly in highly vulnerable communities [11, 12]. This evidence underscores how structural racism and place‐based disadvantage jointly shape perinatal risk.

Our study extends this work by quantifying how racial disparities vary across levels of community vulnerability using county‐level multilevel models and DID estimation. Moving beyond earlier cross‐sectional or aggregate approaches, we demonstrate that disparities in preterm birth, SGA birth, and HDP systematically widen as social vulnerability increases. These findings provide more rigorous evidence that social vulnerability not only raises overall perinatal risk but also amplifies racial inequities through the compounding effects of structural racism [24, 25, 26].

The mechanisms linking social vulnerability to preterm birth are multifactorial. Communities with high SVI scores experience greater chronic stress, environmental hazards, food insecurity, and reduced access to high‐quality prenatal care [11, 12]. These exposures intersect with systemic racism, producing a disproportionate burden for Black birthing individuals. The weathering hypothesis provides a useful framework: chronic exposure to social and structural disadvantage accelerates biological aging and increases allostatic load, thereby heightening susceptibility to adverse pregnancy outcomes [26]. Economic hardship, discrimination, air pollution, substandard housing, limited prenatal care access, and food insecurity each contribute to cumulative physiologic stress and elevate preterm birth risk. Our finding that racial disparities widen as community vulnerability increases is consistent with this model.

The shape of these risk gradients is also informative. Although preterm birth rates rise for both Black and White births as community vulnerability increases, the slope is considerably steeper for Black births—a pattern documented in prior research and attributed to the cumulative burden of structural inequities across the life course [24, 26]. This suggests that improvements in community conditions may yield disproportionately greater benefits for White populations unless accompanied by targeted, equity‐focused interventions that address the underlying drivers of racialized disadvantage [24].

These mechanisms have important policy implications. The SVI, as a publicly available and validated indicator, could serve as a tool to identify high‐risk communities and guide equitable resource allocation. Place‐based interventions such as expanding access to high‐quality prenatal care, reducing environmental exposures, and strengthening housing and food security are essential for mitigating the impact of community vulnerability on preterm birth. Equally critical is ensuring that these efforts are grounded in racial equity and community engagement. Participatory approaches enable policies and programs to reflect the lived realities of those most affected and enhance the likelihood of durable impact [27].

### 4.3 Research implications

Despite growing evidence linking social vulnerability to adverse perinatal outcomes and widening racial disparities, several critical questions remain unanswered. First, while county‐level measures such as the SVI capture broad community disadvantage, it remains unclear which specific structural factors—such as housing instability, food insecurity, or healthcare access—most strongly drive disparities in preterm birth [8, 9, 10]. Second, while some studies have suggested that Medicaid expansion may improve overall birth outcomes, the effects on racial disparities in preterm birth remain inconsistent [12]. For instance, a study in Texas found that Medicaid expansion was associated with a 2.04‐percentage‐point decrease in preterm birth rates among White infants, but did not show significant changes for Black infants [28]. Conversely, a national study indicated that Medicaid expansion was associated with significant reductions in relative disparities in preterm birth rates between Black and White infants [29]. These mixed findings underscore the need for further research to understand the nuanced impacts of Medicaid expansion on racial disparities in preterm birth. Future studies should employ longitudinal and quasi‐experimental designs, such as DID analyses, to assess how the policy influences racial disparities in preterm birth and related outcomes across different states and populations.

### 4.4 Strengths and limitations

Strengths of this study include the use of a large, nationally representative cohort, linkage with a validated community‐level index, and assessment of dose–response patterns across quartiles. Strengths of this study include the use of a large, nationally representative cohort derived from restricted county‐level CDC natality data, enabling granular assessment of population‐level patterns across US counties. By linking these records to a validated community‐level SVI, we were able to capture multidimensional aspects of social disadvantage. The analysis incorporated both categorical and continuous modeling of SVI, including spline‐based sensitivity analyses and secondary analyses based on the severity of preterm birth, which supported the robustness of findings. Furthermore, the application of a DID framework allowed us to quantify disparity gradients across vulnerability quartiles, providing a more nuanced understanding of how community context modifies racial inequities in adverse pregnancy outcomes.

Limitations of this study should be noted. First, reliance on birth certificate data introduces potential for misclassification and underreporting, particularly for conditions such as HDP. The dataset did not distinguish between spontaneous and iatrogenic preterm births, which may have different underlying etiologies and risk factors. Although we used robust statistical methods and adjusted for multiple confounders, residual confounding remains possible, particularly from unmeasured factors such as hospital‐level practices, patient migration, and local or state policy environments. The use of administrative data also limits the granularity of clinical and behavioral variables that may influence outcomes. Analyses were restricted to Black and White birthing populations, limiting generalizability to other racial and ethnic groups. Additionally, county‐level measures of social vulnerability may mask substantial within‐county heterogeneity, particularly in counties characterized by high residential segregation. In such settings, Black and White births may experience systematically different neighborhood environments despite sharing the same county‐level SVI classification, leading to differential exposure misclassification. This limitation may disproportionately attenuate estimates among White births residing in lower‐vulnerability census tracts of high‐SVI counties. Future studies linking natality data to census tract–level measures are needed to evaluate how within‐county inequality modifies racial disparities in preterm birth. Further, SVI includes a domain capturing racial and ethnic minority concentration, which reflects structural vulnerability rather than individual race. While this aligns with our focus on structural disadvantage, results may differ if alternative indices excluding race, such as the Area Deprivation Index or Child Opportunity Index, were used. Because the natality dataset includes only live births, fetal deaths were not captured. If social vulnerability or race is differentially associated with fetal loss, our estimates may be subject to live‐birth bias. Finally, the cross‐sectional design precludes causal inference.

## 5 CONCLUSION

County‐level social vulnerability was associated with higher preterm birth rates and widening Black–White disparities in the United States. These findings underscore that the structural disadvantages embedded in socially vulnerable communities compound but cannot fully account for racial disparities in perinatal outcomes. Addressing these disparities requires policies that extend beyond individual clinical care to address upstream social and structural determinants of health.

## CONFLICT OF INTEREST STATEMENT

The authors declare no conflicts of interest.

## ETHICS STATEMENT

This study qualified for exemption from Institutional Review Board review and adhered to the STROBE reporting guidelines.

## Supporting information



Supporting Information
