# Supplementary material for: The association between social vulnerability and racial disparities in preterm birth
Source: Pregnancy (Hoboken). 2026 Apr 7;2(3):e70279. doi: 10.1002/pmf2.70279 (PMC13344310; doi:10.1002/pmf2.70279)
Supplement: Supplementary file 1 [file PMF2-2-e70279-s001.docx]

Supplemental Online Materials

eFigure 1: Adjusted rates of small-for-gestational-age (SGA) births by Social Vulnerability Index (SVI) quartile and maternal race.

eFigure 2. Adjusted rates of Hypertensive Disorders of Pregnancy (HDP) by Social Vulnerability Index (SVI) quartile and maternal race.

eTable 1. Maternal Characteristics by Social Vulnerability Index (SVI) Quartile and Race.

eTable 2. Crude analyses considering the random intercepts for state and county.

eTable 3. Association Between Severe Preterm Birth and Social Vulnerability Index (SVI) Quartile, Stratified by Race.

eTable 4. Outcomes according to the social vulnerability index and race after excluding small counties

eFigure 1: Adjusted rates of small-for-gestational-age (SGA) births by Social Vulnerability Index (SVI) quartile and maternal race.

This figure displays the adjusted rate of SGA among births to Black and White individuals across SVI quartiles, with higher quartiles representing greater social vulnerability. SGA rates increased with each higher SVI quartile for both racial groups. At every level of vulnerability, Black individuals experienced substantially higher adjusted SGA rates compared with White individuals, and this gap widened in the highest SVI quartile. Error bars represent 95% confidence intervals.

eFigure 2. Adjusted rates of Hypertensive Disorders of Pregnancy (HDP) by Social Vulnerability Index (SVI) quartile and maternal race.

This figure illustrates the adjusted rate of HDP among births to Black and White individuals across SVI quartiles, with higher quartiles indicating greater social vulnerability. HDP increased steadily with rising SVI for Black individuals. In contrast, White individuals had adjusted HDP rates that steadily decreased as SVI increased. Error bars represent 95% confidence intervals.

eTable 1*.* Maternal Characteristics by Social Vulnerability Index (SVI) Quartile and Race

| Characteristic | White | | | | Black | | | | P-value |
| --- | --- | --- | --- | --- | --- | --- | --- | --- | --- |
|  | **SVI Q1** | **SVI Q2** | **SVI Q3** | **SVI Q4** | **SVI Q1** | **SVI Q2** | **SVI Q3** | **SVI Q4** |  |
| N | 328,620 | 462,466 | 491,149 | 391,497 | 27,154 | 73,441 | 119,925 | 228,537 | NA |
| Age (yr) | 30.4 (± 5.2) | 30.0 (± 5.4) | 29.6 (± 5.6) | 29.8 (± 5.6) | 30.2 (± 6.1) | 29.5 (± 6.2) | 28.7 (± 6.2) | 28.4 (± 6.2) | <0.001 |
| GA at delivery (wk) | 38.6 (± 1.7) | 38.6 (± 1.7) | 38.5 (± 1.8) | 38.5 (± 1.8) | 38.3 (± 2.2) | 38.2 (± 2.2) | 38.1 (± 2.3) | 38.0 (± 2.3) | <0.001 |
| Prepregnancy BMI (kg/m^2^) | 28.5 (± 10.8) | 28.6 (± 11.5) | 28.6 (± 11.3) | 28.6 (± 11.5) | 31.4 (± 14.0) | 31.7 (± 14.4) | 31.2 (± 13.3) | 31.6 (± 13.8) | <0.001 |
| Insurance |  |  |  |  |  |  |  |  | <0.001 |
| Medicaid | 65,177 (19.8) | 114,458 (24.7) | 150,006 (30.5) | 127,105 (32.5) | 13,891 (51.2) | 41,260 (56.2) | 77,301 (64.5) | 154,480 (67.6) |  |
| Private | 242,451 (73.8) | 311,984 (67.5) | 297,850 (60.6) | 229,543 (58.6) | 11,605 (42.7) | 27,874 (38.0) | 35,687 (29.8) | 60,437 (26.4) |  |
| Self-pay | 11,284 (3.4) | 18,634 (4.0) | 22,519 (4.6) | 15,515 (4.0) | 613 (2.3) | 1,634 (2.2) | 2,883 (2.4) | 5,592 (2.4) |  |
| Other | 9,708 (3.0) | 17,390 (3.8) | 20,774 (4.2) | 19,334 (4.9) | 1,045 (3.8) | 2,673 (3.6) | 4,054 (3.4) | 8,028 (3.5) |  |
| Infertility treatment | 11,989 (3.7) | 15,538 (3.4) | 12,709 (2.6) | 9,270 (2.4) | 556 (2.1) | 948 (1.3) | 1,097 (0.9) | 1,437 (0.6) | <0.001 |
| Smoking | 13,127 (4.0) | 20,792 (4.5) | 25,953 (5.3) | 15,455 (4.0) | 657 (2.4) | 2,052 (2.8) | 4,027 (3.4) | 5,707 (2.5) | <0.001 |
| Chronic hypertension | 10,391 (3.2) | 14,261 (3.1) | 15,961 (3.3) | 10,989 (2.8) | 1,447 (5.3) | 3,840 (5.2) | 7,219 (6.0) | 12,649 (5.5) | <0.001 |
| Pregestational diabetes | 3,145 (1.0) | 4,537 (1.0) | 5,043 (1.0) | 3,587 (0.9) | 440 (1.6) | 1,309 (1.8) | 1,974 (1.6) | 3,676 (1.6) | <0.001 |

Abbreviations: SVI, Social Vulnerability Index; GA, gestational age; BMI, body mass index; NA (not applicable); SD, standard deviation; Q, quartile.

eTable 2. Crude analyses considering the random intercepts for state and county.

| **Outcome** | **SVI Quartile** | **Black (%, 95%CI)** | **White (%, 95%CI)** | **AME (95% CI)** | **DID (95% CI)** | **Interaction P** |
| --- | --- | --- | --- | --- | --- | --- |
| **Preterm birth** | Q1 | 10.76 (10.25, 11.28) | 7.61 (7.45, 7.76) | 3.16 (2.65, 3.66) | [Reference] | <0.001 |
|  | Q2 | 12.25 (11.82, 12.68) | 7.87 (7.72, 8.02) | 4.38 (3.97, 4.80) | 1.23 (0.57, 1.88) |  |
|  | Q3 | 12.72 (12.37, 13.08) | 8.31 (8.16, 8.47) | 4.41 (4.06, 4.76) | 1.25 (0.64, 1.87) |  |
|  | Q4 | 13.75 (13.43, 14.06) | 8.60 (8.40, 8.80) | 5.15 (4.81, 5.49) | 1.99 (1.38, 2.60) |  |
| **HDP** | Q1 | 11.54 (10.87, 12.20) | 11.59 (11.29, 11.89) | -0.05 (-0.68, 0.58) | [Reference] | <0.001 |
|  | Q2 | 12.14 (11.54, 12.74) | 11.49 (11.18, 11.80) | 0.66 (0.10, 1.21) | 0.71 (-0.13, 1.55) |  |
|  | Q3 | 12.68 (12.17, 13.19) | 11.54 (11.25, 11.82) | 1.14 (0.64, 1.65) | 1.19 (0.38, 2.00) |  |
|  | Q4 | 13.00 (12.46, 13.55) | 10.97 (10.65, 11.30) | 2.03 (1.44, 2.61) | 2.08 (1.22, 2.94) |  |
| **SGA** | Q1 | 13.48 (12.78, 14.18) | 6.50 (6.35, 6.64) | 6.98 (6.28, 7.68) | [Reference] | 0.008 |
|  | Q2 | 15.45 (14.91, 15.99) | 7.23 (7.08, 7.37) | 8.22 (7.70, 8.75) | 1.24 (0.36, 2.11) |  |
|  | Q3 | 16.28 (15.82, 16.73) | 7.45 (7.31, 7.59) | 8.83 (8.37, 9.29) | 1.85 (1.01, 2.68) |  |
|  | Q4 | 17.09 (16.71, 17.47) | 7.88 (7.72, 8.04) | 9.21 (8.80, 9.61) | 2.22 (1.42, 3.03) |  |

**Abbreviations:** SVI, Social Vulnerability Index; AME, Average Marginal Effect; DID, Difference-in-Differences; CI, Confidence Interval; HDP, Hypertensive Disorders of Pregnancy; SGA, Small for Gestational Age. Numbers are only adjusted for the random effects of state and county.

Supplemental Table 3. Association between early preterm birth and social vulnerability index according to race

| Outcome | SVI Quartile | Black, % (95% CI) | White, % (95% CI) | AME (95% CI) | DID (95% CI) | Interaction *P* |
| --- | --- | --- | --- | --- | --- | --- |
| <34 weeks | Q1 | 2.86 (2.65, 3.08) | 1.65 (1.59, 1.70) | 1.22 (1.00, 1.44) | [Reference] | <0.001 |
|  | Q2 | 3.20 (3.06, 3.35) | 1.69 (1.65, 1.74) | 1.51 (1.36, 1.65) | 0.29 (0.03, 0.55) |  |
|  | Q3 | 3.30 (3.18, 3.42) | 1.78 (1.73, 1.83) | 1.52 (1.39, 1.65) | 0.30 (0.05, 0.55) |  |
|  | Q4 | 3.59 (3.48, 3.70) | 1.75 (1.70, 1.81) | 1.84 (1.71, 1.96) | 0.62 (0.37, 0.87) |  |
| <32 weeks | Q1 | 1.82 (1.65, 1.99) | 0.85 (0.82, 0.89) | 0.97 (0.79, 1.14) | [Reference] | 0.001 |
|  | Q2 | 1.96 (1.86, 2.07) | 0.87 (0.83, 0.90) | 1.10 (0.99, 1.20) | 0.13 (−0.07, 0.33) |  |
|  | Q3 | 2.04 (1.94, 2.14) | 0.93 (0.89, 0.96) | 1.11 (1.02, 1.21) | 0.15 (−0.05, 0.34) |  |
|  | Q4 | 2.26 (2.18, 2.35) | 0.91 (0.88, 0.95) | 1.35 (1.26, 1.44) | 0.38 (0.19, 0.57) |  |
| <28 weeks | Q1 | 0.83 (0.71, 0.95) | 0.29 (0.27, 0.31) | 0.54 (0.42, 0.66) | [Reference] | 0.36 |
|  | Q2 | 0.87 (0.80, 0.95) | 0.30 (0.28, 0.32) | 0.58 (0.50, 0.65) | 0.04 (−0.11, 0.18) |  |
|  | Q3 | 0.89 (0.83, 0.96) | 0.33 (0.31, 0.35) | 0.56 (0.50, 0.63) | 0.03 (−0.11, 0.16) |  |
|  | Q4 | 0.98 (0.92, 1.03) | 0.33 (0.31, 0.35) | 0.65 (0.59, 0.71) | 0.11 (−0.02, 0.24) |  |

**Abbreviations:** SVI, Social Vulnerability Index; AME, Average Marginal Effect; DID, Difference-in-Differences; CI, Confidence Interval. Numbers are adjusted for maternal age, body mass index, chronic hypertension, pregestational diabetes, smoking status, insurance type, and the random effects of state and county.

Average marginal effects (AME) represent the absolute differences in severe preterm birth between Black and White individuals within each SVI quartile. Difference-in-differences (DID) estimates quantify how racial disparities change across increasing levels of social vulnerability, with Quartile 1 serving as the reference. Interaction P values indicate whether the association between SVI quartile and severe preterm birth differs by race. Models are adjusted for maternal age, body mass index, chronic hypertension, pregestational diabetes, smoking status, insurance type, and the random effects of state and county.

eTable 4. Outcomes according to the social vulnerability index and race after excluding small counties

| **Outcome** | **SVI Quartile** | **Black (%, 95%CI)** | **White (%, 95%CI)** | **AME (95% CI)** | **DID (95% CI)** | **Interaction P** |
| --- | --- | --- | --- | --- | --- | --- |
| **Preterm birth** | Q1 | 9.44 (8.86, 10.01) | 7.36 (7.11, 7.60) | 2.08 (1.54, 2.62) | [Reference] | <0.001 |
|  | Q2 | 11.08 (10.64, 11.52) | 7.86 (7.61, 8.11) | 3.22 (2.83, 3.61) | 1.14 (0.47, 1.80) |  |
|  | Q3 | 11.16 (10.80, 11.51) | 8.06 (7.83, 8.30) | 3.10 (2.76, 3.44) | 1.02 (0.39, 1.65) |  |
|  | Q4 | 12.23 (11.90, 12.57) | 8.46 (8.20, 8.72) | 3.77 (3.43, 4.12) | 1.69 (1.06, 2.32) |  |
| **HDP** | Q1 | 11.73 (10.56, 12.90) | 11.91 (10.82, 13.00) | -0.18 (-0.99, 0.62) | [Reference] | <0.001 |
|  | Q2 | 12.41 (11.49, 13.32) | 11.85 (11.09, 12.61) | 0.55 (-0.08, 1.19) | 0.74 (-0.27, 1.75) |  |
|  | Q3 | 12.91 (12.28, 13.54) | 11.74 (11.19, 12.28) | 1.18 (0.66, 1.69) | 1.36 (0.40, 2.32) |  |
|  | Q4 | 13.53 (12.82, 14.24) | 11.49 (10.95, 12.04) | 2.04 (1.32, 2.75) | 2.22 (1.17, 3.27) |  |
| **SGA** | Q1 | 12.64 (11.92, 13.36) | 6.42 (6.18, 6.66) | 6.22 (5.50, 6.95) | [Reference] | 0.41 |
|  | Q2 | 14.09 (13.64, 14.55) | 7.04 (6.83, 7.26) | 7.05 (6.61, 7.49) | 0.83 (-0.02, 1.67) |  |
|  | Q3 | 14.36 (14.01, 14.71) | 7.05 (6.87, 7.24) | 7.31 (6.94, 7.67) | 1.08 (0.27, 1.89) |  |
|  | Q4 | 14.73 (14.47, 15.00) | 7.38 (7.21, 7.55) | 7.35 (7.04, 7.66) | 1.13 (0.35, 1.91) |  |

**Abbreviations:** SVI, Social Vulnerability Index; AME, Average Marginal Effect; DID, Difference-in-Differences; CI, Confidence Interval; HDP, Hypertensive Disorders of Pregnancy; SGA, Small for Gestational Age. Numbers are adjusted for maternal age, body mass index, chronic hypertension, pregestational diabetes, smoking status, insurance type, and the random effects of state and county.

Models are adjusted for maternal age, body mass index, chronic hypertension, pregestational diabetes, smoking status, insurance type, and the random effects of state and county.

Counties with <100 births in either racial group were excluded.
